# Ten simple rules on how to write a standard operating procedure

**DOI:** 10.1371/journal.pcbi.1008095

**Published:** 2020-09-03

**Authors:** Susanne Hollmann, Marcus Frohme, Christoph Endrullat, Andreas Kremer, Domenica D’Elia, Babette Regierer, Alina Nechyporenko, on behalf of Cost Action CA15110

**Affiliations:** 1 Potsdam University, Potsdam, Germany; 2 SB Science Management UG (haftungsbeschränkt), Berlin, Germany; 3 Technical University of Applied Sciences, Wildau, Germany; 4 ITTM Information Technology for Translational Medicine, Esch-sur-Alzette, Luxemburg; 5 Institute for Biomedical Technologies, National Research Council, Bari, Italy; 6 Kharkiv National University of Radio Electronics, Kharkiv, Ukraine; Dassault Systemes BIOVIA, UNITED STATES

## Abstract

Research publications and data nowadays should be publicly available on the internet and, theoretically, usable for everyone to develop further research, products, or services. The long-term accessibility of research data is, therefore, fundamental in the economy of the research production process. However, the availability of data is not sufficient by itself, but also their quality must be verifiable. Measures to ensure reuse and reproducibility need to include the entire research life cycle, from the experimental design to the generation of data, quality control, statistical analysis, interpretation, and validation of the results. Hence, high-quality records, particularly for providing a string of documents for the verifiable origin of data, are essential elements that can act as a certificate for potential users (customers). These records also improve the traceability and transparency of data and processes, therefore, improving the reliability of results. Standards for data acquisition, analysis, and documentation have been fostered in the last decade driven by grassroot initiatives of researchers and organizations such as the Research Data Alliance (RDA). Nevertheless, what is still largely missing in the life science academic research are agreed procedures for complex routine research workflows. Here, well-crafted documentation like standard operating procedures (SOPs) offer clear direction and instructions specifically designed to avoid deviations as an absolute necessity for reproducibility.

Therefore, this paper provides a standardized workflow that explains step by step how to write an SOP to be used as a starting point for appropriate research documentation.

## Introduction

Nowadays, digital technologies are integral to how knowledge is produced and shared, and science is organized. Data availability is a critical feature for an efficient, progressive, and, ultimately, self-correcting scientific ecosystem that generates credible findings and has become a relevant element of scientific integrity [1]. However, the anticipated benefits of sharing are achieved only if data are of reliable quality and reusable [2,3]. Despite this need, it has been shown that fewer than one-third of (biomedical) papers can be reproduced in general [4,5]. Further studies showed that suboptimal data curation, unclear analysis specification, and reporting errors could impede reuse and analytic reproducibility, undermining the utility of data sharing and the credibility of scientific findings [6–9]. Standard operating procedures (SOPs) for industrial processes to achieve efficiency, quality, and uniformity of performance have existed since a long time ago. SOPs ensure that the user operates following consistent processes that meet best practice standards. Moreover, the use of SOPs ensures that processes are reviewed and updated regularly and that researchers inside and outside the same group or institute are enabled to reproduce or reuse results to enlarge the study or for other studies. Despite their importance, the need for the use of standards in life science research has emerged as crucial for the quality and reproducibility of research findings only in recent years [9]. The question of data quality and reproducibility poses new scientific and societal challenges for individual researchers, universities, scientific organizations, infrastructure facilities and funders, and the broader society [10]. The scientific community started to talk about a crisis of reproducibility and its dramatic impact on the economy and credibility of the research system [9,11] around 2016. Many community-driven initiatives such as the Research Data Alliance (RDA) [12] and the European Commission prompted a series of initiatives in support of the scientific community to cope with problems related to the need of new tools and strategies to improve the harmonization of standard initiatives [13–14] and the implementation of standards in the daily research work to improve research quality and data reuse. Since then, many steps forwards have been made in different fields of biological research [15–20].

There are recommendations providing guidelines for maintaining reproducible results by just applying simple rules. Those rules include the use of error annotations for produced data that are viable for evaluating the impact and credibility of single data associated with the generated results. Furthermore, it is essential to use annexes when research project results are published. Annexes aid the traceability of findings and the reproducibility of performed experiments and can be linked to the aforementioned error classifications. Input files, along with information of the applied software versions, perfectly fit into annexes and play a pivotal role for reproducing results. Finally, adopting these simple rules in the routine research practice within an SOP format aid the transparency of results and exert a decisive impact on scientific reproducibility [21]. Against this background, the implementation of a minimal quality assurance (QA) system as a systematic approach to review practices and procedures is inherent logical [22]. QA systems enable the users to identify possible improvements and errors and provide a mechanism for their use, for example, by developing and deploying a failure mode and effects analysis (FMEA) [23]. The basis of each quality system is a high-quality record providing a string of documents for the verifiable origin and quality of data. Also, the general documentation improves the traceability and transparency of research findings to prove the reliability of results. Such quality control systems should be based on and be in line with good laboratory practices (GLP), well-defined and validated protocols, and comprehensive SOPs [24–25]. The advantages of implementing SOPs in the daily workflow of academic researchers might not be immediately obvious and enlighten everyone. At first, it seems to be unnecessary and avoidable extra work. Indeed, without appropriate training, the setup of an SOP is time-consuming and does not appear to be a relevant asset. However, because each SOP describes one procedure only and not a series of complex procedures the efforts to be done remain feasible. For this reason, we provide you here with “10 Simple Rules on How To Write an SOP” that will enable you to produce a reliable and verifiable set of your research data.

## Results

### The Ten Rules

**Fig 1:**
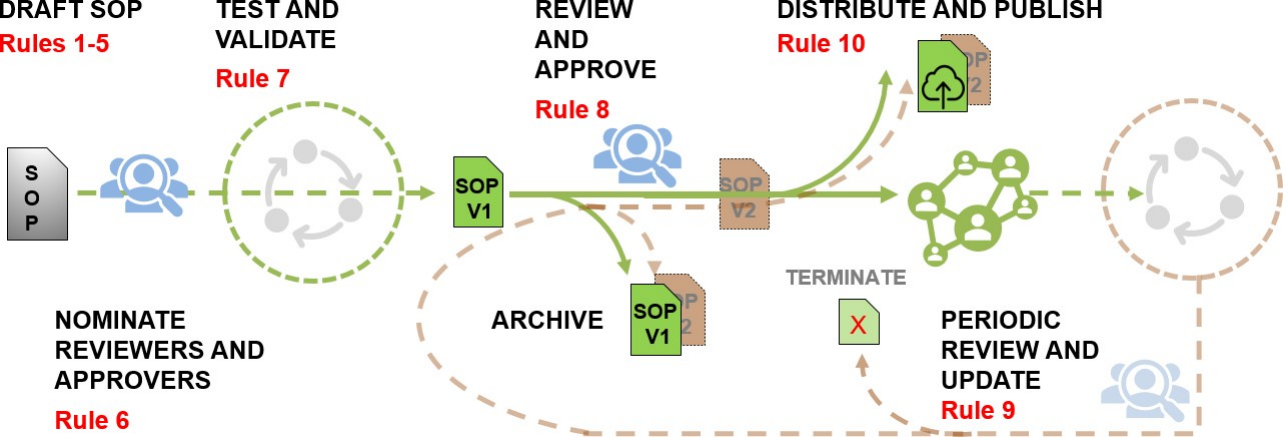
SOP workflow. Workflow of SOP development, its implementation, and monitoring.

Fig 1 demonstrates the workflow of SOP writing along the line from its preparation, validation, and approval to its implementation and follow-up processes, which will be detailed in the following 10 rules.

### Rule 1: Knowing when to write an SOP

SOPs are always needed when critical processes or workflows need to be repeated in a reproducible way or when defined procedures are obliged by compliance guidelines. In other words, SOPs are vital instruments for maintaining consistent quality. Hence, every research institution which faces such processes, compliance requirements or has demand for quality should be encouraged to develop new or follow existing SOPs. Crosscheck within your institution for templates. There is no need to reinvent the wheel. Often templates are existing in your institution, but their existence might not be well communicated. Contact your data or quality manager for information. There also often exists a shared team server or cloud, containing all internal SOPs being accessible for each appropriate member or user, respectively. If your institute or department does not have official templates, ask other groups if they already have SOPs in use. If so, your colleagues should be happy to share their knowledge with you. If there is no template available, you have a pioneering role. In this case you can run a quick online search for SOP templates to be used as a starting point. It is wise to involve the technicians of your lab as well as the leader of your group or department and the quality manager and/or data manager of your institution. The initial step is now to draft a cover sheet and discuss the content of the SOP with the relevant stakeholders (reviewers and approvers) of your institution (Rules 3 and 6). Depending on the peculiarity of research you can choose an appropriate SOP format or develop your own. The resulting unique template can then be further used by all members of your group, department, or institution and by your wider community of practice.

### Rule 2: Write the introduction: describe the purpose (the why)

An essential aspect of any SOP is introduction and purpose forming. The introduction section specifies the need and capability of the procedures for the research environment in which the process is being established. Identify the specific reason you decided to write your SOP. Which specific process do you address? Which specific procedures will be covered, and which are not covered? Clarifying what the specific focus of your document is will facilitate the use of your SOP by your colleagues. Richie Norton writes, “Simplicity is complex. It's never simple to keep things simple. Simple solutions require the most advanced thinking” [26]. The way you frame the information describing the process matters. Consider "less is more." Trying to tackle too much at once will mostly only lead to confusion. Represent the complex aspects of a process in a way to make it as simple as possible to make it also understandable even for users who are not from your field.

### Rule 3: Set up the document structure

Every single SOP should consist of three sections:

1. The cover page
2. The sequence of steps or tasks (metadata) for the given procedure (Rule 4)
3. A list of references and definitions (Rule 5)

### The cover page

The cover page represents a control block used to house the document control information required to configure management and compliance standards.

It should contain the following information (Fig 1):

- administrative information about the institution and/or department
- a title that clearly identifies the activity or procedure
- an SOP identifier (ID) number (or versioning) with its category (Table 1)
- page number and total number of pages of the SOP
- the date of issue (or of versioning)
- possible safety instructions
- the names of individuals who prepared and approved the SOP
- the name of the reviewers, including the date of the review
- a description of the purpose and field of application
- the name and function of the author
- the name and function of the approver

**Table 1:** Makeup of the documents cover page.

STANDARD OPERATING PROCEDURE					
**Your Institution**		**Your Logo**			
**Short Title, ID**		**Page**		**of**	
**Title**					
**Version**		**Created on**			
**Status**		**Related SOP**			
**Safety instruction**		**Category**			
**Purpose and field of application**					
**Author**					
	**Function, name, signature**				
**Approved by**					
	**Function, name, signature**				

The SOP’s cover page should provide information about traceability of the SOP, including information about the purpose and scope, its author, issuing, any changes made, and its status (draft, approved, revised, or removed).

There are standardized abbreviations to describe elements of the respective SOP on the cover page. Table 2 provides a list of typical abbreviations for an SOP category.

**Table 2:** List of abbreviations used for allocation of the SOP’s category.

Short name	Meaning
F	fundamental
M or METH	analytical method
P	procedure to carry out a special investigation (project)
PROT	protocol
ORG	organizational document
WS	worksheet
*A* or *APP*	apparatus
SOP; *P* or *PROJ*	procedure to carry out a special investigation (project)
*PROT*	protocol describing a sequence of actions or operations
*PERS*	to describe personnel matters
*RF*	registration form (e.g., chemicals and samples)

It is recommended to use a digital object identifier (DOI) for the identification of the SOP in accordance with ISO 26324:2012 [27].

Each following page should have a heading and/or footing note mentioning (Table 3)

- administrative information about the institution and/or department
- short title of the SOP
- page number and total number of pages of the SOP
- date of approval and/or version number

A version number can be assigned in accordance with semantic versioning [28] recommendations as guidelines used in source control systems like GitHub [29].

The presentation of dates, including date of issue, versioning, date of review, and approval should be in accordance with the format specified in ISO 8601 [30].

**Table 3:** Makeup template for the header of SOP instruction pages.

STANDARD OPERATING PROCEDURE					
**Short Title, ID**		**Page**		**of**	
**Title**					
**Version**		**Created on**			
**Status**		**Related SOP**			
**Procedure**					

All SOP pages should have an identifier referring to the cover page and consist of administrative information about the institution and/or department, the short title of the SOP, the page number out of the total number, the date, and approval and/or version number.

The final step in creating your SOP template should involve a note on the styles, fonts, and margins that you intend to use. You can download a full template SOP at Zenodo as a writable PDF [31].

### Rule 4: Fill in the content

#### The procedure

Start at page 2 to compile the metadata. In this step you describe the activities and the sequence of steps or tasks for the given procedure. Always consider the aim of the SOP; this will help you to focus on the specific procedure you describe. However, an SOP may contain multiple SOPs provided that each one is cited by number and full information is provided. Every user should be able to understand your work instructions. As George Orwell said, "Good [writing] is like a windowpane" [32]. Consider the knowledge and skills of potential users and the level of detail to present the process description. Long preambles should be avoided. Work instructions should follow a single style and follow a stepwise process strictly. Balance the level of detail, avoid unnecessary specification (e.g., “blue-cap tubes”) and alternatives; if alternatives are necessary, explain what dictates which action. Ensure consistency in terms of terminology, layout, media, and method and, as much as possible, avoid polysyllabic words, complex sentences, jargon, acronyms, or too many terms (without explaining them). To facilitate handling of the different data types and formats within the same workflow, the impact of such diversity to the usability of data and metadata should be minimized. Consider the different work cultures and different circumstances within people’s work and explain and describe the how and what to do. To optimize the structure of you SOP.

- break the process into sections
- break the sections into specific steps
- number the steps or add bullet points for clarity.

Describe each task in detail, including timeframes and tools required to complete assignments and achieve expected outcomes.

### Rule 5: References and definitions: Specify tools required for the task

The workflow elements may involve different types of resources and tools. Depending on a particular task, the selection of tools can also comprise modeling and simulation tools, data repositories, and compiler construction tools. Do not forget to add relevant references. Reference materials can include normative documents, instructions, and standards as well as research papers, graphical material, photographs, and even different SOPs. Provide all definitions of terms and abbreviations that are to be used in the procedure. Harmonize and align with standard terminologies used within your field. These should be provided as an annex.

### Rule 6: Set up responsibilities and nominate reviewers and approvers

Reviewers should be nominated and appointed responsible for each particular task. These persons should have appropriate scientific knowledge and expertise as well as experience in the field. Usually, the initial SOP-author(s) is/are responsible for monitoring and reviewing the SOP. They will ensure that the SOP reflects the tasks described in the document. Open discussion on controversial aspects related to the SOP should be allowed among reviewers to guarantee a proper revision. Changes to SOPs should preferably be made by the creator. Each change needs again to be reviewed and approved by the responsible persons and by an increase of the revision number (Rule 8). The team of reviewers should agree on a timeframe to monitor and align the current state-of-the-art SOP.

### Rule 7: Test with a colleague: Perform training

Congratulations! By reaching this step you wrote your first SOP. Now it is time to check if the SOP is clear and understandable for all potential users. Hence ask a colleague (it can be a researcher or a technician) to read the SOP and, if feasible, execute a test run. Ask the test person to be constructive and critical and wait for his or her feedback or questions. Consider that the test person should be able to run the experiment without any support. Do not interfere while testing to receive authentic feedback and to avoid falsifying the result.

Once written, it is essential that all staff is appropriately trained with and familiarized in the use of the new SOP. This can easily be integrated within the annual safety instructions or as part of a running seminar series. Retraining should be conducted regularly but also when there is a change in SOPs. Ensure that showing attendance at training is documented.

### Rule 8: Review and approve

Once your SOP is tested, make it available for the reviewers’ team for a final check. Once substantial comments are received, discuss them with your colleagues to ensure a successful outcome and that the SOP is clear for everybody. Include all relevant edits to improve the document. Repeat the procedure until the SOP is agreed by all stakeholders and, subsequently, send the document to the quality manager for approval afterwards. The final document should be sent to the assigned SOP approvers, who will sign it. In case an SOP is out of date due to the introduction of new technologies or changes in the organizational structure, the SOP should be terminated, labelled (out of date), and archived for traceability. The new and valid version should be distributed. All valid versions should be stored and accessible in a folder at a central location. By this, you ensure that valid and approved SOPs exist and every employee receives the information necessary for their work. To keep track of any deviations from existing SOPs, the establishment and deployment of an exception log might be a powerful instrument. This log should ideally entail a documentation of any deviation, the reason for the deviation, the outcome, any troubleshooting which has been applied, and resolutions and appropriate communication. Regarding the latter, issues or reasons for deviations as well as necessary changes or modifications in the SOP should be discussed with and signed by all involved stakeholders and supervisors, respectively [33].

### Rule 9: Update document: Specify validation and periodic review date

Do not forget to review and update an SOP regularly to keep it up to date and useful for current and future use. Your SOP should be validated and reviewed periodically to improve the document and reflect any changes that have been made or are necessary. All changes should be entered into a revision form, which comprises version number, change data, reason and description for change, reviewers’ data, and signatures. The revised or updated document should be shared immediately with all respective users, while clarifying that the former SOP is outdated. The SOP compliance maintenance could be easily implemented by using an electronic lab notebook (ELN), which might keep track of all deviations automatically, dependent on software and functions.

In this case, ensure the interoperability of used formats to enable export of the data into other systems. Furthermore, regular mandatory user training should be set up for the most important SOPs to ensure compliance.

### Rule 10: Publish

It's all very good to have work instructions, but what is their value if they are only available in your office when the users who need them are somewhere else? The people performing the work should have easy access to the work instructions anytime and anywhere, most easily accomplished by setting up a secured team server or cloud on which all latest versions are uploaded. To add value to your SOP, make it available to a broad user community you should upload your SOP also in open access public repositories such as Zenodo [34], SEEK [18], OpenAIRE [35], FAIRsharing [36–37], or another.

## Conclusion

Nowadays digital technologies and advanced computational methods are an integral part of daily laboratory practice. To best manage the generated data and avoid reproducibility issues, scientists need to implement FAIR data principles, suitable DMPs, and appropriate documentation. The lack of reproducibility within laboratory research discourages successful implementation of the wide-spread adoption of research results in the scientific community. One way to improve it is to provide consistency and traceability of existing standards and laboratory practices that are achievable with precise and clearly written SOPs.
